# Clinical Approaches of Whole Body Vibration Exercises

**DOI:** 10.1155/2018/9123625

**Published:** 2018-11-21

**Authors:** Mario Bernardo-Filho, Redha Taiar, Borja Sañudo, Trentham Furness

**Affiliations:** ^1^Laboratório de Vibrações Mecânicas e Práticas Integrativas, Departamento de Biofísica e Biometria, Universidade do Estado do Rio de Janeiro, RJ, Brazil; ^2^Department of Sport Science, Université de Reims Champagne-Ardenne, Reims, France; ^3^Departamento de Educación Física y Deporte, Universidad de Sevilla, Sevilla, Spain; ^4^Australian Catholic University, School of Nursing, Midwifery and Paramedicine, Victoria, Australia

The importance of clinical approaches to promote/maintain health, wellbeing, and quality of life in the context of disease is salient. These clinical approaches can vary in simplicity from basic improvement of healthy lifestyle choices involving, for example, physical activity, to complex combinations of pharmacological, surgical, and multidisciplinary clinician intervention [1]. Importantly there is the need, if possible, to offer simple and effective nonpharmacological interventions with limited side effects that contribute to the quality of life and independence of the individuals. During rehabilitation, when patients are vulnerable and less likely to be able to independently participate in even basic interventions, the efficacy of low skill physical activities is critical [2].

The use of Whole-Body Vibration Exercise (WBVE) in clinical settings is becoming ever-present in the literature. As a relatively simple and low skill demand mode of physical activity, WBVE has been used in athletic and aged cohorts. As benefits of WBVE have begun to emerge, it has progressively been applied to various clinical populations. The purpose of this special issue it to present scientific considerations about WBVE, as there is a lack of synthesis about the safe and effective prescription of WBVE as a mode of rehabilitation. There is a need for clinicians to be provided with clear recommendations about the parameters of WBVE such as frequency (Hz), amplitude (mm), duration (min), and gravitational load (g) that will be tolerated by suboptimal health populations during rehabilitation and practice [3–6]. A further purpose was to invite studies that used randomised controlled, case controlled, and placebo controlled studies that clearly described the WBVE intervention that will allow identical replication in other clinical settings. Studies that attempt to describe adverse effects (if apparent) and studies that demonstrate the valid parameters of the WBV platform (including the direction: vertical or oscillating) and biomechanical parameters of the WBVE (such as frequency, the peak to peak displacement, and acceleration) with clinical populations were preferred. As in some occupational activities, individuals are exposed to whole body vibration, and this subject was also considered important to be in this special issue. Papers that provide recommendations for the prescription of the WBVE to improve outcomes relative to the clinical subgroups were also included.

Review papers which describe the current state of the art and papers involving different types of studies involving WBVE are available in this special issue that has the intention to provide information to a better and safe use of the WBVE. Papers involving studies with WBVE including (i) neurophysiological basis, (ii) the effect on the pain due several diseases, (iii) evaluations about the consequences in different populations with various diseases (metabolic, neurological), or health individuals to the rehabilitation, the promotion of the health and the quality of life and (iv) in young and older adults are available in this special issue. Combination of therapies involving intervention with mechanical vibration and mirror therapy is also present in this special issue.

Therefore, this special issue addresses a topic of great interest, such as the beneficial effects of WBVE in the working context, especially for office workers who spend most of their time in sitting position. Moreover, it would be interesting to see the acute effects of this therapy on inflammatory markers in people with chronic obstructive pulmonary Disease on the control of glucose levels in Type II diabetes, the postural stability in physically active elderly women, or even whether individuals with stroke or traumatic brain injury can profit from this method. It seems that these studies suggest that WBVE may be a complementary therapy in gait rehabilitation and different functional outcomes of the patients (i.e., calf muscle spasticity in patients with poststroke hemiplegia). As some positive results can be found, this special issue can contribute to promoting further research in this area, testing the feasibility and efficacy of using WBVE in other populations or comparing different WBVE parameters.

Another intention of this special issue was to highlight to clinicians that WBVE can be an effective addition to traditional rehabilitation interventions to improve clinical and functional aspects of recovery. Clinicians need to be provided with specifics of WBVE prescription such as duration of WBVE exposure, rest intervals, type and length of intervention, and body posture during WBVE.

Considering all the challenges to organize this special issue, we thank the Hindawi office for the confidence and all the authors that have contributed to try to bring a useful issue involving the proper use of the WBVE, contributing thus to the scientific evidence, the dissemination of knowledge about the benefits, and the care necessary for the safe use of WBVE.
